# Slipins: ancient origin, duplication and diversification of the stomatin protein family

**DOI:** 10.1186/1471-2148-8-44

**Published:** 2008-02-11

**Authors:** Jasper B Green, J Peter W Young

**Affiliations:** 1 Department of Biology, University of York, UK

## Abstract

**Background:**

Stomatin is a membrane protein that was first isolated from human red blood cells. Since then, a number of stomatin-like proteins have been identified in all three domains of life. The conservation among these proteins is remarkable, with bacterial and human homologs sharing 50 % identity. Despite being associated with a variety of diseases such as cancer, kidney failure and anaemia, precise functions of these proteins remain unclear.

**Results:**

We have constructed a comprehensive phylogeny of all 'stomatin-like' sequences that share a 150 amino acid domain. We show these proteins comprise an ancient family that arose early in prokaryotic evolution, and we propose a new nomenclature that reflects their phylogeny, based on the name "slipin" (stomatin-like protein). Within prokaryotes there are two distinct subfamilies that account for the two different origins of the eight eukaryotic stomatin subfamilies, one of which gave rise to eukaryotic SLP-2, renamed here "paraslipin". This was apparently acquired through the mitochondrial endosymbiosis and is widely distributed amongst the major kingdoms. The other prokaryotic subfamily gave rise to the ancestor of the remaining seven eukaryotic subfamilies. The highly diverged "alloslipin" subfamily is represented only by fungal, viral and ciliate sequences. The remaining six subfamilies, collectively termed "slipins", are confined to metazoa. Protostome stomatin, as well as a newly reported arthropod subfamily slipin-4, are restricted to invertebrate groups, whilst slipin-1 (previously SLP-1) is present in nematodes and higher metazoa. In vertebrates, the stomatin family expanded considerably, with at least two duplication events giving rise to podocin and slipin-3 subfamilies (previously SLP-3), with the retained ancestral sequence giving rise to vertebrate stomatin.

**Conclusion:**

Stomatin-like proteins have their origin in an ancient duplication event that occurred early on in the evolution of prokaryotes. By constructing a phylogeny of this family, we have identified and named a number of orthologous groups: these can now be used to infer function of stomatin subfamilies in a meaningful way.

## Background

Human stomatin (hstomatin) was first identified as an integral membrane protein in human red blood cells [1-3]. It has since been shown to be expressed in many cell types and organisms, although hstomatin function remains unclear [4]. Loss of stomatin in humans is associated with a condition called overhydrated hereditary stomatocytosis, in which the red blood cells leak Na^+ ^and K^+ ^ions [5], although the hstomatin gene is not mutated in these patients [6]. Other human proteins showing high similarity to human stomatin (> 50 %) have also been described. Human stomatin-like protein-2 (hSLP-2) is a 39 kDa, widely expressed, oligomeric, peripheral membrane protein that associates with the spectrin-actin cytoskeleton in the red cell [7]. It has recently been shown to be overexpressed in a variety of human tumours [8], being one of the 16 most upregulated proteins in superinvasive cancer cells, although its function is again unknown [9]. Human stomatin-like protein-3 (hSLP-3), an olfactory neuronal protein [10], shares 84 % similarity with hstomatin and is important for the function of skin mechanoreceptors in the mouse [11]. Podocin is 73 % similar to hstomatin and, like stomatin, is raft associated [12]. Podocin is expressed exclusively in the kidneys, where it is localised to the membrane of podocytes; these are specialised cells involved in the ultrafiltration of blood [13]. Mutations in the podocin gene (NPHS2) result in nephritic syndrome, in which protein appears in the urine; the end stage of this condition is renal failure [14]. The final member of this putative family is human stomatin-like protein-1 (hSLP-1), which differs from the other stomatin proteins in that it is a bipartite protein that contains a stomatin-like region fused to a non-specific lipid transfer protein [15].

Stomatin-like proteins are not confined to humans. Work on *Caenorhabditis elegans *has identified at least nine proteins showing similarity to human stomatin. One of these, UNC-24, is necessary for the movement of another protein from the endoplasmic reticulum to the cell membrane [16] and has recently been shown to share a common ancestor with hSLP-1 [17]. The apicomplexan parasite *Plasmodium falciparum *contains a stomatin-like protein that co-localises with invasion-associated rhoptry organelles and is involved in the formation of the invasion vacuole during infection of red blood cells [18]. Of particular interest to us is the prokaryotic group of stomatin-like proteins. These were first identified by You and Borthakur [19] who showed, through a mutagenesis screen, that a stomatin-like protein was involved in the competitiveness of *Rhizobium etli *for nodulation of the roots of *Phaseolus vulgaris*. The widespread distribution of stomatins and their associated diseases strongly suggests that their biological functions are of great importance, yet to date these remain unclear. If we are to understand the function of human stomatins by studying these proteins in other organisms then it is important that we can distinguish sequences that have evolved by speciation (orthologues) from those that have evolved by duplication (paralogues): to achieve this end we need a stomatin family phylogeny. So far, stomatin family evolution has always been considered in the context of a superfamily involving stomatins, prohibitins, flotillins and HflK/C proteins [20] and plant disease response genes [21]. However, more recently this superfamily concept has been revisited, and is now regarded to have little phylogenetic support [22]. It is therefore likely that similarity among members is a result of convergent evolution and not shared ancestry.

In this paper we have chosen to undertake a phylogenetic analysis of stomatin-like proteins only. Our results reveal an intriguing story of ancient origin, duplication and diversification of stomatin family members and identifies candidate organisms that should be used when attempting to understand stomatin function outside of primate systems.

## Results

### Two Different Origins of Eukaryotic Stomatins

Blast searches for hstomatin and hSLP-2 revealed highly similar proteins in prokaryotes, both in archaea and bacteria (Table 1). Proteins showing high similarity to hSLP-2 were also identified in eukaryotes, although SLP-2 distribution is confined to specific organisms among the fungi and protista and only becomes well represented in plant and metazoan lineages. In contrast, proteins showing high similarity to hstomatin were only found in prokaryotes, fungi, ciliates and animals. SLP-3 and podocin proteins were again found to be restricted to specific metazoan species (Table 1).

**Table 1:** A selection of stomatin subfamilies.

Group	Species Representative	Stomatin	Slipin-3* [SLP-3]	Podocin	Slipin-4**	Paraslipin* [SLP-2]
Primates	*Homo sapiens*	NP_004090.4 (100)	NP660329.1 (100)	NP_055440.1 (100)	-	NP_038470.1 (100)
Rodents	*Mus musculus*	NP_038543.1 (87)	NP_694796.1 (91)	NP_569723.1 (87)	-	NP_075720.1 (94)
Birds	*Gallus gallus*	XP_415401.2 (94)	XP_425632.2 (76)	XP_422265.2 (62)	-	XP_430510 (53)
Amphibians	*Xenopus tropicalis*	-	NP_989344.1 (68)	-	-	NP_001004808.1 (82)
Teleosts	*Danio rerio*	NP_571833.1 (83)	-	NP_001018155.1 (44)	-	NP_957325.1 (81)
Insects	*Drosophila melanogaster*	NP_729018.1 (67)	-	-	NP_573357.1 NP_996512.1	NP_611853.2 (65)
Crustaceans	*Daphnia pulex*	JGI_45422 (71)	-	-	JGIest_193650 JGI_45422	JGIest_204990 (68)
Nematodes	*Caenorhabditis elegans*	NP_508902.3 (69)	-	-	-	NP_492517.2 (64)
Annelids	*Capitella sp*. I	JGIest_172850 (71)	-	-	-	JGIest_225245 (64)
Choanoflagellates	*Monosiga brevicollis*	-	-	-	-	JGIest_ 32235 (62)
Fungi	*Schizosaccharomyces pombe*	-	-	-	-	NP_596756.1 (53)
	*Debaryomyces hansenii*	XP_457231.1 (30) Ω	-	-	-	XP_457451.1 (58)
Plants	*Arabidopsis thaliana*	-	-	-	-	NP_200221.1 (55)
	*Oryza sativa*	-	-	-	-	NP_001061036.1 (48)
Stramenopiles	*Phytophthora ramorum*	-	-	-	-	JGI_71814 (57)
Kinetoplastids	*Trypanosoma cruzi*	-	-	-	-	XP_808902.1 (47)
Apicomplexa	*Plasmodium falciparum*	-	-	-	-	NP_473293.1 (48)
Ciliates	*Tetrahymena thermophila*	XP_001033024.1 (35) Ω				XP_001027964.1 (44)
Diplomonads	*Giardia lamblia*	-	-	-	-	**-**
Entamoeba	*Entamoeba histolytica*	-	-	-	-	**-**
						
Archaea (Crenarchaeota)	*Aeropyrum pernix*	NP_148418.2 (52) ‡	-	-	-	**-**
Archaea (Euryarchaeota)	*Pyrococcus abyssi*	NP_126340.1 (49) ‡	-	-	-	NP_127238.1 (42)
Bacteria	*Burkholderia thailandensis*	YP_440000.1 (48) ‡	-	-	-	YP_442572.1 (51)
	*Neisseria meningitidis*	-	-	-	-	NP_274245.1 (49)

Accession numbers or JGI protein IDs of the proposed subfamily members are listed in each column. If genomes contained multiple copies of the same protein, the sequence with the highest BLAST score was selected. Numbers in parentheses indicate % amino acid identity to the human protein in the column heading. – indicates that no match was found in that species. * indicates the new subfamily name, [names in square brackets are previously used names], ** indicates a newly reported subfamily, ‡ = eoslipin [p-stomatin], Ω = alloslipin. Note that slipin-1 [SLP-1] proteins are not included but are highly diverged members of the stomatin protein family.

**Figure 1:**
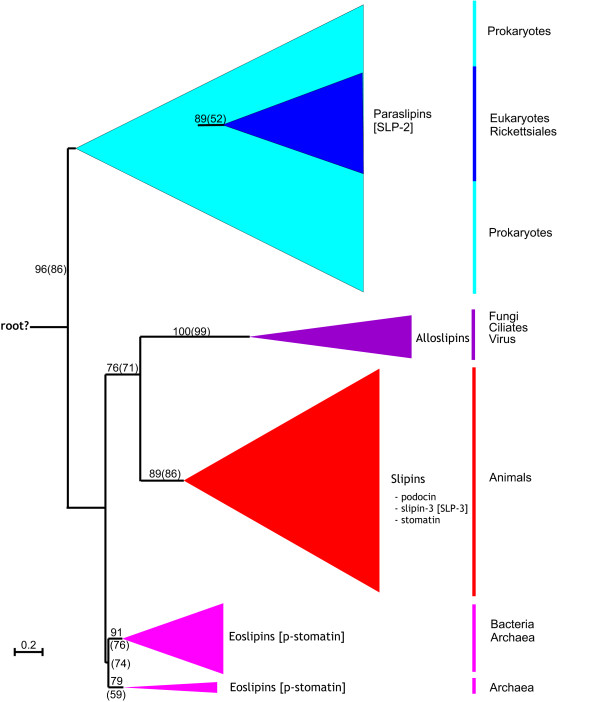
**Maximum likelihood (ML) phylogeny of stomatin family members**. Neighbour Joining (NJ) and ML trees were constructed with the same 149 amino acid alignment. 100 ML and 1000 NJ bootstraps were performed. Percentage bootstrap values are shown for the major groups that were recovered in either the ML or NJ (in parentheses) phylogeny. The virus sequence comes from the *Acanthamoeba polyphaga *mimivirus. The scale bar indicates the number of amino acid substitutions per site. The length of each triangle corresponds to longest branch length, whilst the height represents the number of taxa. Names in square brackets represent previous nomenclature. The full tree is available as additional file 1: Figure A1. Note the partitioning of the family into at least four major groups termed here paraslipins, eoslipins, slipins and alloslipins, with a long internal branch between paraslipins and the other subfamilies where we have tentatively located the root. Accession numbers available in Additional file 2.

Using the amino acid sequences retrieved by the blast searches, a phylogeny was created using both neighbour joining (NJ) and maximum likelihood (ML) methods to get an overview of the relationships among the various family members (Figure 1). In both cases an essentially congruent topology was generated, with the phylogeny identifying at least four distinct groups: SLP-2 proteins, p-stomatin proteins, a group uniting fungi, ciliates and an *Acanthamoeba polyphaga *mimivirus sequence, and a group containing only animal subfamilies. Bootstrap support for the internal branches is mostly good. The branching order of eukaryotic subfamilies in Figure 1 is addressed later by constructing smaller trees with longer alignments. P-stomatin proteins form a paraphyletic group in Figure 1, but this is poorly supported (< 75 %) and is in conflict with the NJ phylogeny, where p-stomatin proteins were recovered as a well supported group (74 %). Archaeal SLP-2 proteins form a paraphyletic group with the inclusion of bacteria, whilst bacterial SLP-2 proteins form another paraphyletic group with the inclusion of eukaryotes. Both ML and NJ methods support the rickettsia-eukaryotic SLP-2 group, although their position within this clade is much less resolved.

**Figure 2:**
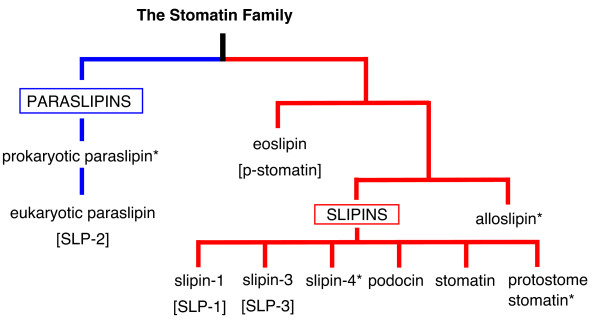
**Stomatin subfamilies**. This diagram illustrates the various subfamilies of stomatin-like proteins recovered by phylogenetic analysis in Figures 1-5, and our proposed nomenclature. Names in [] represent previous nomenclature if a new term is being proposed, whilst * indicates a newly reported subfamily.

From Figure 1 it is clear that not all stomatin family proteins are equally related. Indeed, SLP-3 is much more stomatin-like than SLP-2, yet current nomenclature does not reflect this. To make this distinction clear we propose to split the stomatin family into four major groups: slipins (**s**tomatin-**li**ke **p**rote**ins)**, eoslipins ('eo' from Greek *eos *meaning dawn), alloslipins ('allo' from Greek *allos *meaning other) and paraslipins (**para**logous **s**tomatin-**li**ke **p**rote**ins**). The phylogenies we present here reveal slipin subfamilies to include stomatin, podocin, slipin-1 (not shown, previously SLP-1), slipin-3 (previously SLP-3), slipin-4 and protostome stomatin. The former SLP-2 proteins become paraslipins whilst p-stomatin proteins become eoslipins. From this point forwards, the stomatin subfamilies will be named according to this new nomenclature, which is clarified in Figure 2.

### Paraslipin Subfamily Phylogeny

**Figure 3:**
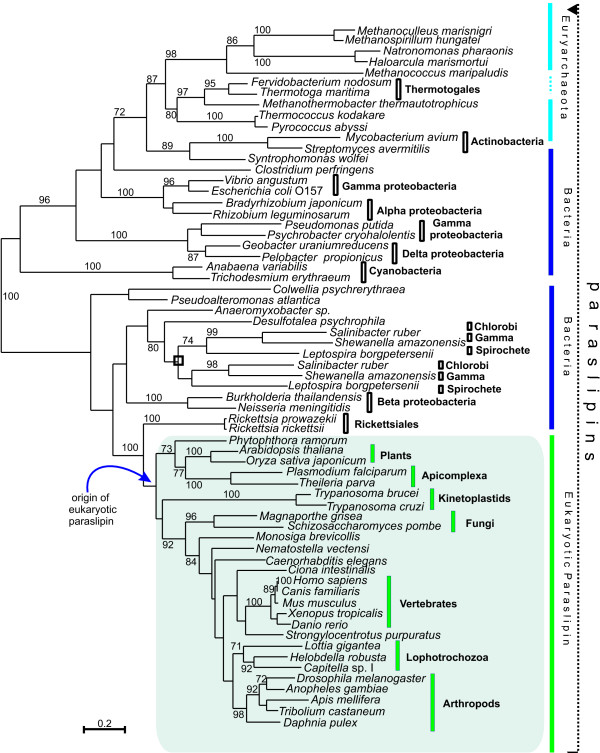
**Maximum likelihood tree of paraslipin proteins**. The phylogeny was based on a 272 amino acid alignment. Numbers on the branches show percentage bootstrap occurrence of nodes in 100 replicates. Only values > 70 are shown. The scale bar indicates the number of amino acid substitutions per site.  indicates the clade uniting Chlorobi, Gammaproteobacteria and Spirochetes with two parallel phylogenies. Note the position of Rickettsiales as the sister group to the eukaryotic clade. Accession numbers available in Additional file 2.

In order to obtain a better-resolved phylogeny of paraslipin proteins, an alignment excluding all other subfamilies was constructed and this was used to build a maximum-likelihood phylogeny (Figure 3). The evolution of prokaryotic paraslipins deserves detailed consideration, but we confine ourselves here to the briefest outline as context for the eukaryotic paraslipins. The phylogenetic tree shows two major clades, both of which contain bacterial species. The upper clade contains both archaeal and bacterial species, with archaea forming a clade that appears to branch within bacteria. Although related species are generally grouped together, such as the Cyanobacteria and the Firmicutes, the tree is inconsistent in several respects with the consensus phylogeny based on ribosomal and other core genes [23] suggesting prokaryotic paraslipin has encountered various gene duplication and lateral transfer events.

The lower paraslipin clade contains both bacterial and eukaryotic species. Representatives of the gamma Proteobacteria, Chlorobi and Spirochetes each have two, divergent copies of prokaryotic paraslipin suggesting an early duplication event. A very significant feature of the lower group is the strong support (100 %) for a monophyletic group containing Rickettsiales and eukaryotic paraslipins, with Rickettsiales forming the sister group to the eukaryotic clade. This suggests a possible mitochondrial origin of eukaryotic paraslipins. Within eukaryotes we see a mostly well-resolved phylogeny with many of the major taxonomic groups being recovered as monophyletic. As expected, fungi form the sister group to metazoa with plants and protists falling outside of this Opisthokont clade [24]; the ecdysozoan group of insects and nematodes is not supported, but neither is it significantly contradicted.

### Protostome stomatins

**Figure 4:**
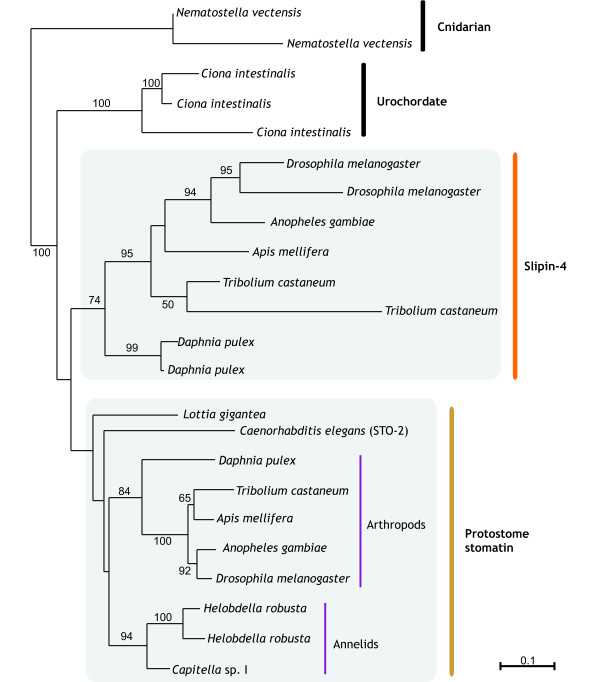
**Maximum likelihood tree of protostome slipins**. Phylogenies were based on a 239 amino acid alignment. The scale bar indicates the number of amino acid substitutions per site. 100 bootstraps were performed and values ≥ 50 are shown on each branch. Cnidarian sequences were included to root the phylogeny whilst the sea urchin *Ciona intestinalis *sequences were included to allow comparison with Fig. 5. Accession numbers available in Additional file 2.

**Figure 5:**
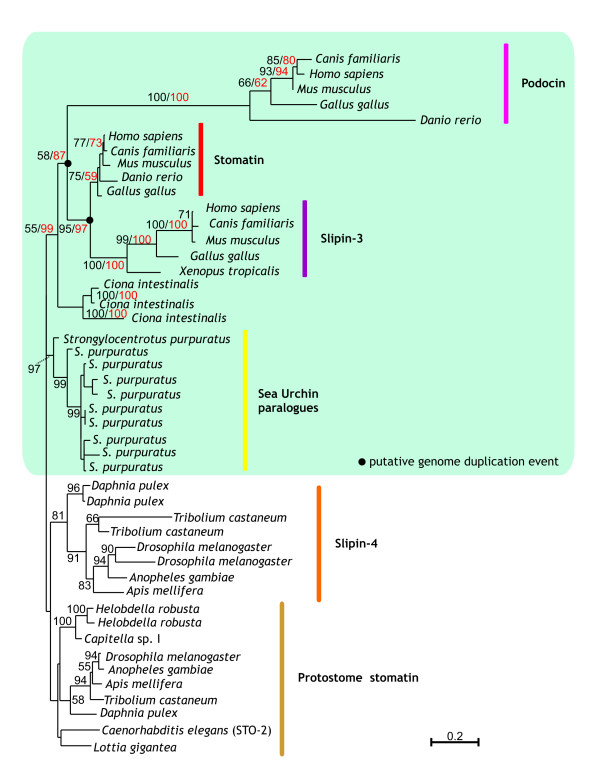
**Maximum likelihood phylogenies of metazoan slipins**. The full tree is based on a 227 amino acid alignment of chordate, echinoderm and protostome sequences. 100 bootstraps were performed and values ≥ 50 are shown in black on each branch. A separate phylogeny, shown by the green box, was constructed from a longer, 252 amino acid alignment of only chordate and echinoderm sequences. 100 bootstraps were performed and values ≥ 50 are shown in red on each branch. Accession numbers available in Additional file 2.

Much of the work on the stomatin family has concentrated on a group of *C. elegans *proteins that were identified in a screen to identify touch-insensitive mutants [25]. With this in mind, we wanted to construct a comprehensive phylogeny of invertebrate stomatins. Within invertebrates it is evident that a large number of gene duplication events have occurred. Constructing a single phylogeny of all invertebrate slipin sequences proved problematic due to a large number of long-branched, species-specific paralogues. To overcome this problem, smaller phylogenies of closely related taxa were first constructed to identify orthologous groups; these were subsequently combined to give the phylogeny in Figure 4. *Ciona intestinalis *sequences were included to allow comparison with Figure 5, whilst cnidarian sequences were used to root this phylogeny. Within Figure 4 we see two main clusters. One such group contains arthropods, annelids, the mollusc *Lottia gigantea *and the nematode *C. elegans*, and we tentatively name this group protostome stomatin as a consequence of it members and short branch lengths, despite the low bootstrap for this clade. The other group concerns only arthropods and appears much more divergent than protostome stomatin, but nevertheless recovers the same insect phylogeny; this group we have named slipin-4.

### Vertebrate Stomatins

From Figure 1 it was apparent that many stomatin subfamilies arose within the vertebrate lineage. To try to understand the origin and evolution of vertebrate sequences, two separate phylogenies were constructed and are shown in Figure 5. The first phylogeny was based on an alignment of chordate and echinoderm sequences, and this was rooted with slipin-4 and protostome stomatin sequences. Although poorly supported, chordate and protostome sequences form separate groups.

To gain more information about the duplication events that occurred within the vertebrate portion of our tree, a vertebrate-specific phylogeny were constructed that allowed for a longer alignment (Figure 5, shaded box). Stomatin sequences from the echinoderm *Strongylocentrotus purpuratus *and the urochordate *Ciona intestinalis *were included so that an approximate time frame for the origin of these subfamilies could be established. The vertebrate-specific phylogeny recovers the same topology as seen if slipin-4 and protostome sequences are included but, in addition, provides strong support (87 %) for the monophyly of vertebrate subfamilies with the *Ciona intestinalis *sequences forming the sister group. Within vertebrates there are three well-supported monophyletic groups. Podocin proteins form the most basal vertebrate group and are clearly quite divergent from the other subfamilies, as judged by their long branch. The other two clades group stomatin and slipin-3 proteins into two well-resolved clusters. In each case the three clades support congruent phylogenies, although not all vertebrates were found to have all proteins (Table 1). However, recently derived paralogues may be substituting for the function of missing genes.

### Domain Characterisation

**Figure 6:**
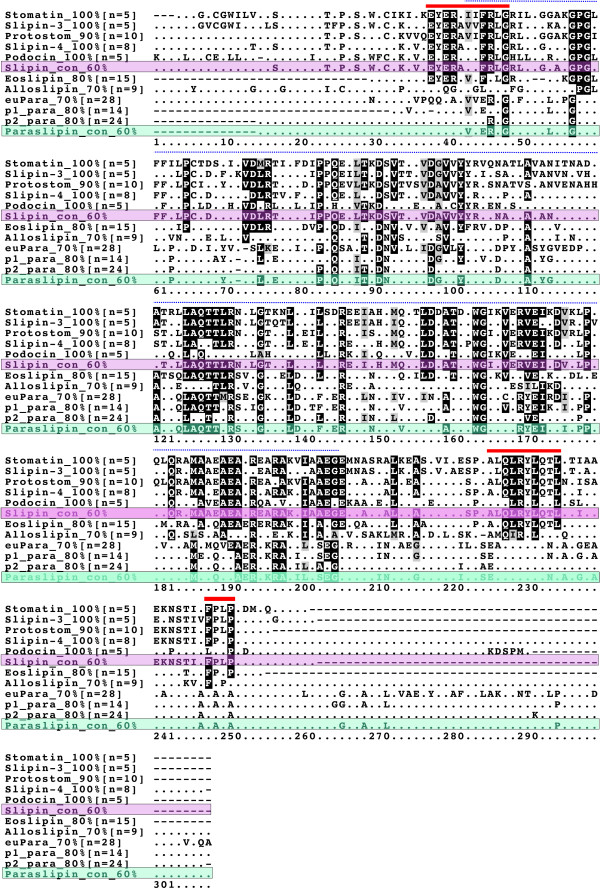
**Alignment of stomatin subfamily consensus sequences**. Consensus sequences (100-70 %) were generated by selecting and aligning (ClustalX) subfamily members identified in monophyletic groups in Figures 1-4. The shading threshold was set to 0.6. Identical residues have a black background and similar residues have a grey background. A 60 % slipin (pink) and paraslipin (green) consensus sequence is also displayed. p2_para refers to prokaryotic paraslipins present in the upper clade in Figure 3, whilst p1_para refers to prokaryotic paraslipins present in the lower clade with eukaryotic paraslipins (euPara). The dotted line above the sequences shows the region shared by all stomatin family members, whilst the solid red line indicates regions shared by slipins and eoslipins to the exclusion of alloslipins. n = the number of sequences used to generate each consensus. – indicates the position of a gap, indicates an unconserved amino acid.

In order to make a meaningful functional assignment to a protein family it is important to characterise the ancestral and derived motifs. To achieve this end, consensus sequences were generated and aligned for each of the proteins identified in monophyletic groups in Figures 1, 2, 3, 4 (Figure 6). The alignment supports the premise that these proteins are members of the same family, as judged by the length of the alignment (150 aa) and the degree of shared conservation. Paraslipin proteins are quite different from the other family members and lack many of the conserved motifs that are shared by the other subfamilies. In terms of sequence conservation, eoslipin sequences most resemble slipin subfamilies and share at least three conserved motifs (red line in Figure 6) to the exclusion of alloslipins. Interestingly, many of the major difference between the stomatin subfamilies occur within the C-terminus region; for example, all podocin proteins can be characterised by a unique five amino acid motif (KDSPM) present in their C-terminus, whilst paraslipins share an AxAxA motif.

## Discussion

Our analyses suggest that the stomatin family is a sound concept, with all its members showing high levels of sequence conservation over a region of 150 amino acids. Eukaryotic stomatin proteins have two independent prokaryotic origins: one gave rise to all eukaryotic paraslipin proteins, whilst the other gave rise to the remaining subfamilies (alloslipin, slipin-1, slipin-3, slipin-4, protostome stomatin, stomatin and podocin).

**Figure 7:**
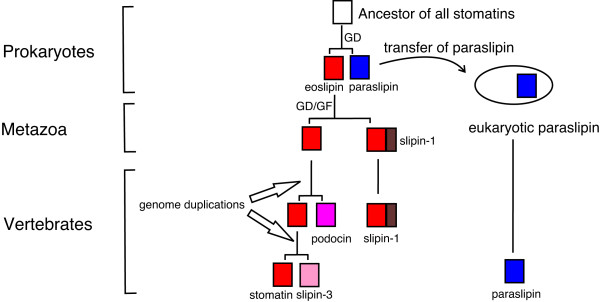
**Simplified hypothesis for the origin, duplication and divergence of vertebrate stomatin subfamilies**. An ancestral stomatin gene, possibly present in the last universal common ancestor, duplicated to give rise to eoslipin and prokaryotic paraslipin. Prokaryotic paraslipin was transferred into eukaryotes during the acquisition of the mitochondrion. Eukaryotic slipins probably evolved from eoslipin, which we assume was present in the last common ancestor of all eukaryotes. Within metazoa, slipin-1 arose from a gene duplication (GD) event involving a stomatin-like gene which subsequently fused (GF) with a sterol carrier domain (Edqvist and Blomqvist 2006). Podocin and slipin-3 arose from two further duplications of an ancestral stomatin-like gene that might have occurred during the two whole genome duplications in early vertebrate evolution.

The stomatin family first arose in prokaryotes (Figure 1). It is supposed that paraslipin and eoslipin are ancient paralogues that evolved from an ancestral sequence, possibly present in LUCA (last universal common ancestor) (Figure 7). Evidence for their homology lies in their sequence similarity (Figure 6) and their genomic organisation. Green *et al*. (2004) showed a conserved genomic association between eoslipin and a serine protease. Within prokaryotic genomes the gene encoding paraslipin has a conserved genomic association with a gene encoding only the C-terminus of the serine protease [26]. It is therefore probable that these two gene clusters share a common ancestor and arose by duplication. The ancientness of this duplication is supported by the observation that eoslipin and paraslipin proteins, as well as their gene partners, are present in both archaea and bacteria, with the bipartition that separates eoslipins and paraslipins being one of the most internal branches in our phylogeny (Figure 1). Although eoslipins are more similar in sequence to stomatins than to paraslipins, we avoid the term 'prokaryotic stomatins' because they are not exclusively related to the stomatins. Indeed, since the root of the tree is uncertain, we are unable to show formally that eoslipins are the sister group of the slipins rather than of the paraslipins.

Figure 3 presents strong support for the transfer of paraslipin (lower clade, Figure 3) into eukaryotes from a rickettsia-like proteobacterium. The source could plausibly have been the progenitor of the mitochondrion [27]. This hypothesis is further supported by the observation that paraslipin is present within the rat mitochondrial proteome [28] and shows a significant decrease in expression in mitochondria devoid of DNA [29]. It is also interesting to note that the only protist species we found not to encode paraslipin were the amitochondriates *Giardia lamblia *and *Trichomonas vaginalis *and the distantly related *Entamoeba histolytica *[30,31]. Once acquired by eukaryotes, paraslipin evolved with very little gene duplication and became taxonomically widespread (Table 1, Figure 3).

The eukaryotic slipin and alloslipin subfamilies probably evolved from a common ancestor shared by archaea and eukaryotes, to the exclusion of bacteria, although our phylogeny is too poorly resolved at present to support such a hypothesis. The discovery of alloslipins is an important finding in our quest to understand stomatin family phylogeny, as these are the first eukaryotic slipin-like sequences identified outside metazoa. However, despite detailed searches of protist and basal metazoan genomes, we have been unable to resolve the currently bizarre taxonomic distribution of alloslipin proteins. Within metazoa, slipins have been subjected to numerous gene duplication events, and at least one gene fusion event with a sterol carrier protein that occurred prior to the divergence of protostomes and deuterostomes [17] and gave rise to SLP-1, now named slipin-1. Within protostomes, there are at least two other subfamilies (Figure 4). One of these groups includes arthropods, molluscs, annelids and a nematode, uniting the protostomes into a monophyletic group, which we have named protostome stomatin to reflect the short branches and phylogenetic range. The topology of Figure 5 suggests that slipin-4 and protostome stomatin may in fact be paralogues that arose before ecdysozoans and lophotrochozoans diverged; following this duplication event slipin-4 was lost from most taxa.

Within vertebrates, we see the origin of podocin, slipin-3 and stomatin proteins, and we propose that these arose as a result of two gene duplication events (Figure 7). The inclusion of *Danio rerio *sequences within all three vertebrate groups suggests both duplication events occurred before the teleost/tetrapod split. The placement of the sea squirt sequences as the sister group to this clade dates the time of divergence to after the chordate/urochordate divergence. If we accept the loss of a paralogue, this phylogeny supports the two whole genome duplication events that are proposed to have occurred prior to the origin of vertebrates [32,33], accounting for the origin of slipin-3, podocin and stomatin. Whilst there appears to have been little sequence divergence in vertebrate stomatin sequences, podocin and, to a lesser extent, slipin-3 have undergone significant sequence evolution (Figures 5 and 6) making it likely that they are functioning in a distinctly different manner to other family members.

## Conclusion

The goal of this study was to provide a conceptual framework within which to study this family. This can be used to improve understanding of stomatin function in humans and to identify relevant homologs to investigate subfamily function. Whilst it is likely that slipin-3, podocin and alloslipin proteins are functioning in new ways (as judged by the long branches leading to these groups), it is not clear whether hstomatin has retained its ancestral function. The lack of any significant divergence within this clade, the conservation of protein length and the sequence conservation, all suggest that vertebrate stomatin may indeed be functioning in a similar way to protostome stomatin and possibly eoslipin. The conservation of motifs (Figure 6) suggests a shared mechanism among these subfamilies, although the downstream effects might be very different. The placement of Rickettsiales as the sister group to eukaryotic paraslipins (Figure 3) suggests that alphaproteobacteria may serve as a relevant system to investigate human paraslipin function.

It seems clear that the stomatin-like proteins have their origin in an ancient duplication event that occurred early on in the evolution of prokaryotes. A high degree of conservation implies that they have important functions, though these remain almost completely unknown. By constructing a phylogeny of this family, we have identified and named a number of orthologous groups. Investigation of many different organisms could potentially contribute to an understanding of stomatin-like proteins, and we hope that our analysis will make it easier to describe and interpret such studies.

## Methods

### Database Searches

The human stomatin amino acid sequence [NCBI:NP_004090.4] was used to search the National Centre for Biotechnology Information (NCBI) non-redundant (nr) database using BLASTP with default settings [34]. The query was restricted to eukaryotes as organisms. To identify prokaryotic stomatin-like proteins, the human stomatin and SLP-2 amino acid sequences were used to search the NCBI nr bacterial and archaeal databases. Sequences with an E-value of < 10^-14 ^were retrieved in FASTA format and saved. Blasting with human stomatin was sufficient to retrieve all stomatin, podocin and SLP-3 proteins. SLP-1 proteins were not retrieved as the presence of the sterol carrier domain limits the alignment length (for a review of SLP-1 phylogeny see Edqvist and Blomqvist 2006). To further explore the distribution of slipins and paraslipins in key eukaryotic genomes, BLASTP and tBLASTn searches were performed against the eukaryotic genomes of the Joint Genome Institute (JGI) using both human stomatin and *Tetrahymena thermophila *[NCBI:XP_001033024.1] alloslipin sequences with default settings.

### Protein Alignment and Phylogenetic Analysis

Retrieved sequences were checked by aligning them with their query sequence using ClustalX 1.83 [35] with the following parameters: gap penalty = 10, gap extension penalty = 0.10. The Gonnet series protein weight matrix was used in the ClustalX alignment. Sequences that failed to align or contained significant gaps (> 50 aa) were deleted. Checked sequences were re-aligned using ClustalX and a preliminary distance Neighbour Joining (NJ) tree [36] was produced for prokaryotic and eukaryotic proteins to determine the number and composition of subgroups, with 1000 bootstrap pseudoreplicates performed. From this initial tree, sequences from well supported monophyletic groups were selected for the various phylogenetic analyses, realigned and edited using BioEdit [37] to remove any ambiguously aligned positions. Where organisms contained multiple copies of the same protein, the protein with the best BLAST score to the search query was chosen. To limit the problems associated with long branch attraction, we removed divergent species-specific paralogues that failed to form orthologous groups. The resulting alignment was then used to construct a NJ tree (ClustalX 1.83, n = 1000) and a maximum likelihood (ML) phylogenetic tree using PHYML [38]. For ML analysis the JTT substitution matrix was used for calculation of the amino acid substitutions [39]. A discrete-gamma distribution with four categories was used to account for variable substitution rates among sites. The gamma distribution parameter was estimated by PHYML. A BIONJ distance tree was used as the starting tree to be refined by the maximum likelihood algorithm. The robustness of the tree was determined by bootstrapping using 100 repetitions. Various subgroups were then selected and used to build further maximum likelihood trees as described above. All trees were displayed using NJ plot [40] except Figure 1 which was viewed using TreeView [41].

### Generation of Consensus Sequences

Sequences from monophyletic groups identified by ML analysis were aligned using ClustalX. BioEdit was then used to create a 70–100 % consensus sequence for each group depending on the number of taxa. X was used to represent a non-consensus position. Consensus sequences were then aligned using ClustalX with default settings and viewed using BOXSHADE [42] with the identity shading threshold set at 0.6.

## Authors' contributions

JBG carried out the analysis and drafted the manuscript. JPWY contributed to the interpretation, to the nomenclature, and to revision of the manuscript. Both JBG and JPWY have read and approved the final version of this manuscript.

## Supplementary Material

Additional file 1 File in PDF format showing the full phylogeny from which Figure 1 is derived. Click here for file

Additional file 2 File in Excel spreadsheet format giving details of sequences used in Figures 1, 3, 4 and 5. All alignments are available upon request from JBG. Click here for file
