# Supplementary figures and images for: Slipins: ancient origin, duplication and diversification of the stomatin protein family
Source: BMC Evol Biol. 2008 Feb 11;8:44. doi: 10.1186/1471-2148-8-44 (PMC2258279; doi:10.1186/1471-2148-8-44)

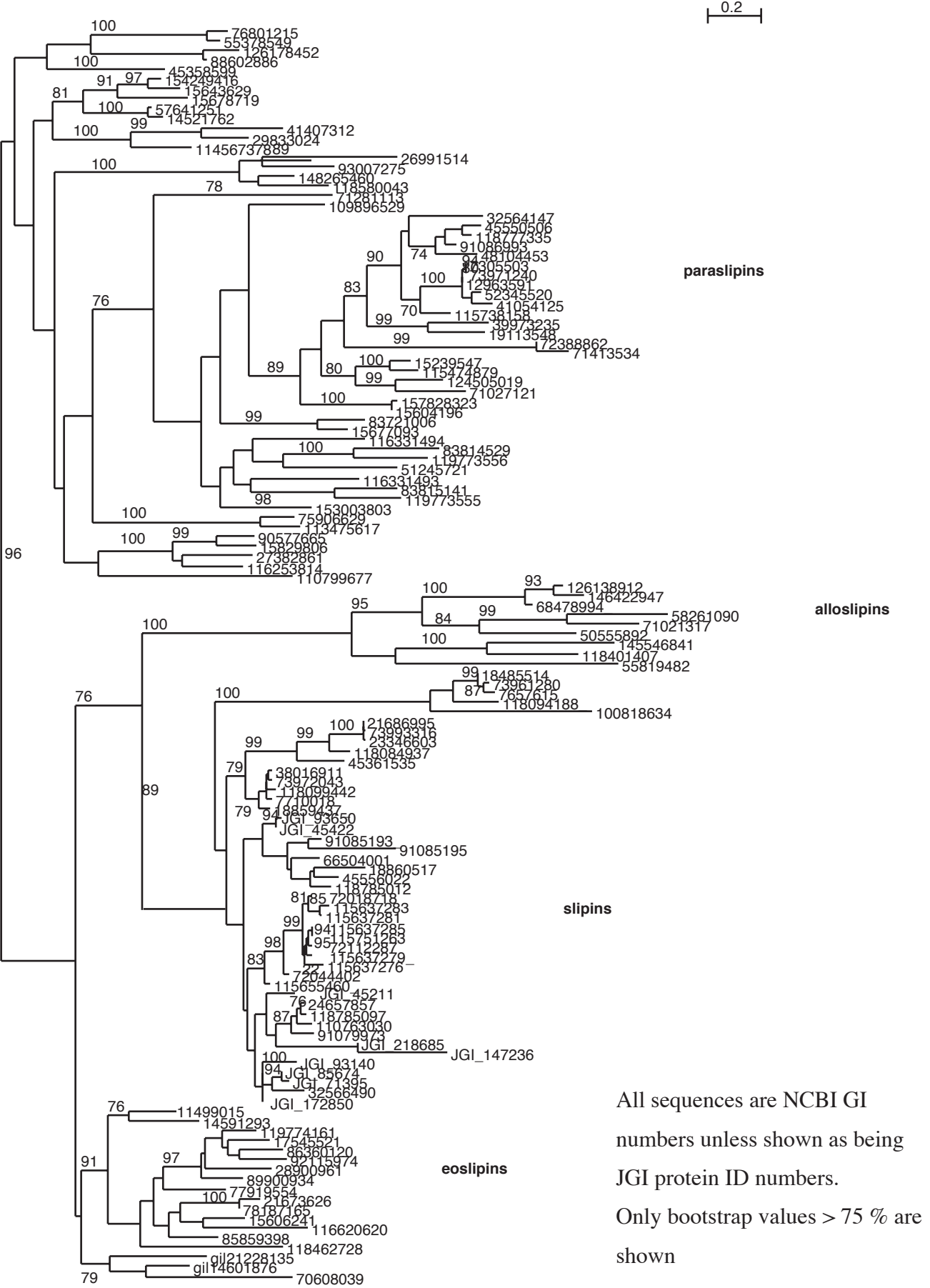

Supplement: Additional file 1 — File in PDF format showing the full phylogeny from which Figure 1 is derived. [file 1471-2148-8-44-S1.pdf]
